# Analysis of new retrogenes provides insight into dog adaptive evolution

**DOI:** 10.1002/ece3.5620

**Published:** 2019-09-06

**Authors:** Xiang Gao, Yan Li, Adeyinka A. Adetula, Yu Wu, Hong Chen

**Affiliations:** ^1^ Center Laboratory Renmin Hospital of Wuhan University Wuhan China; ^2^ Department of Infectious Diseases Zhongnan Hospital of Wuhan University Wuhan China; ^3^ Key Laboratory of Agricultural Animal Genetics, Breeding, and Reproduction Huazhong Agricultural University Wuhan China; ^4^ Oilfield Community D-1-902 Wuhan China; ^5^ Department of Scientific Research Renmin Hospital of Wuhan University Wuhan China

**Keywords:** adaptive evolution, gene movement, new genes, on and off the X chromosome, retrogene

## Abstract

The origin and subsequent evolution of new genes have been considered as an important source of genetic and phenotypic diversity in organisms. Dog breeds show great phenotypic diversity for morphological, physiological, and behavioral traits. However, the contributions of newly originated retrogenes, which provide important genetic bases for dog species differentiation and adaptive traits, are largely unknown. Here, we analyzed the dog genome to identify new RNA‐based duplications and comprehensively investigated their origin, evolution, functions in adaptive traits, and gene movement processes. First, we totally identified 3,025 retrocopies including 476 intact retrogenes, 2,518 retropseudogenes, and 31 chimerical retrogenes. Second, selective pressure along with ESTs expression analysis showed that most of the intact retrogenes were significantly under stronger purifying selection and subjected to more functional constraints when compared to retropseudogenes. Furthermore, a large number of retrocopies and chimerical retrogenes that occurred approximately 22 million years ago implied a burst of retrotransposition in the dog genome after the divergence time between dog and its closely related species red fox. Interestingly, GO and pathway analyses showed that new retrogenes had expanded in glutathione biosynthetic/metabolic process which likely provided important genetic basis for dogs' adaptation to scavenge human waste dumps. Finally, consistent with the results in human and mouse, a significant excess of functional retrogenes movement on and off the X chromosome in the dog confirmed a general pattern of gene movement process in mammals which was likely driven by natural selection or sexual antagonism. Together, these results increase our understanding that new retrogenes can reshape the dog genome and provide further exploration of the molecular mechanisms underlying the dogs' adaptive evolution.

## Graphical abstract

We identified a large number of retrogenes and chimerical retrogenes that originated about 22 million years ago after the divergence time between dog and its closely related species red fox. We found that new retrogenes had expanded in glutathione biosynthetic/metabolic process which likely provided important genetic basis for dogs' adaptation to scavenge human waste dumps. We also found a biased movement on and off the X chromosomes, which was likely driven by natural selection or sexual antagonistic.

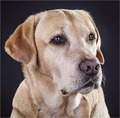

## 1 INTRODUCTION

Since the age of Darwin, biologists have been following an essential question: How do organisms evolve from their common ancestor to a rich variety of species? The origin and subsequent evolution of novel genes have been taken into account as a major contributor to adaptive evolution (Kaessmann, 2010). Novel genes provide important genetic novelties associated with biological diversity in organisms (Chen, Krinsky, & Long, 2013) and significantly contribute to the evolution of lineage‐specific or species‐specific phenotypic traits (Chen, Zhang, & Long, 2010). The molecular processes that can generate new genes mainly include de novo origin, exon or domain shuffling, gene duplication, retrotransposition, TE domestication, gene fission or fusion, gene lateral transfer, and so on (Chen et al., 2013). These mechanisms also cooperate in creating a new gene (Chen et al., 2013; Kaessmann, 2010; Long, Betran, Thornton, & Wang, 2003; Long, VanKuren, Chen, & Vibranovski, 2013).

Retrotransposition is a special RNA‐based duplication mechanism in which transcribed and spliced mRNA is occasionally reverse transcribed and integrated into a new DNA locus to form a retrogene (Betran, Thornton, & Long, 2002). Retrogenes have long been regarded as nonexpressed pseudogenes and evolutionary dead ends due to the lack of regulatory sequences such as promoters. However, extensive structural variations in retrocopies have been speculated as “evolutionary seeds” for the evolution of new genes with novel functions if they casually acquired new regulatory elements or coding sequences by expression (Pan & Zhang, 2009). Retrotransposition is an important mechanism of gene duplication and has produced a large number of functional genes in mammalian genomes (Marques, Dupanloup, Vinckenbosch, Reymond, & Kaessmann, 2005). Recent studies have revealed that a substantial number of “processed genes” or “retrogenes” with novel functions are derived from the mRNA of various intron‐containing genes (Chen et al., 2012; Long & Langley, 1993; Parker et al., 2009; Rosso et al., 2008). For example, *Jingwei*, a new chimeric retrogene only in *Drosophila teissieri* and *Drosophila yakuba*, plays an important role in the metabolism of recruitment pheromones and juvenile hormones (Long & Langley, 1993; Zhang, Yang, Long, Li, & Dean, 2010). Sphinx, a *D. melanogaster‐specific* noncoding RNA gene by retrotranscription, has an impact on male courtship behaviors (Dai et al., 2008). The hominoid‐specific *CDC14Bretro* gene is subjected to a short period of intense positive selection in the African ape ancestor 7–12 Mya and leads to rapid redistribution from microtubules to a new cellular location (Rosso et al., 2008). For RNA‐based duplication pairs, ancestral genes and newly originated retrogenes are easily identified by inspecting their gene structures. Because the ancestral gene contains multiple protein‐coding exons, the retrogene contains a single protein‐coding exon. Therefore, retrogenes provide a convenient and feasible resource to investigate the origin and evolution of new genes.

**Figure 1:**
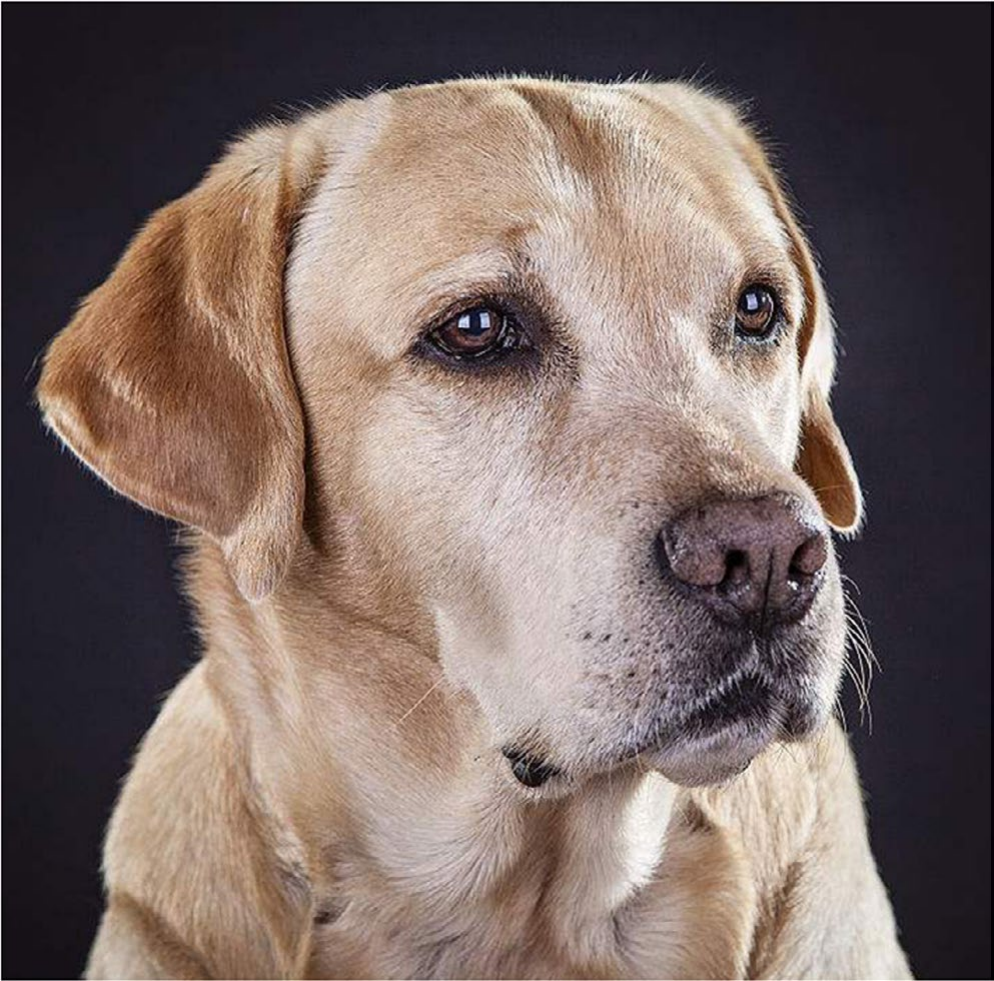
The Labrador retriever

The dogs occupy a special niche in mammalian genomics because they provide important evolutionary information within the mammal. Existing dog breeds show great phenotypic diversity for morphological, physiological, and behavioral traits (Lindblad‐Toh et al., 2005). The dogs are also the first organism that humans domesticated before any plant and any other animal. Previous evidence has revealed that ancient dogs gradually separated from other gray wolves and were involved into humans' life as early as 20,000–40,000 years before present (YBP; Figure 1; Botigue et al., 2017; Skoglund, Ersmark, Palkopoulou, & Dalen, 2015). Constantly following human migration, dogs' diversity has been greatly influenced in many aspects such as breeding, migration, hybridization, invasion, and decimation or assimilation of local populations. The domestic dogs, then, play important roles for geneticists in studying genes and sequence‐level variations which are associated with morphology, susceptibility to simple and complex diseases, and behavior (Malmstrom et al., 2008). For example, genetic variants of IGF1 contribute to a small size phenotype associated with domestication (Sutter et al., 2007). A series of genetic variations have been reported to be associated with the phenotype of coat color in the dog (Berryere, Kerns, Barsh, & Schmutz, 2005; Clark, Wahl, Rees, & Murphy, 2006; Karlsson et al., 2007). A variant in β‐defensin 103 (CBD103), which was previously associated with immune function, results in the dominant phenotype of black coat color in the dog (Candille et al., 2007). SLC1A2 gene is significantly associated with aggression toward strangers (Takeuchi et al., 2009). Interestingly, the expression of a newly originated retrogene fibroblast growth factor 4 (fgf4) is strongly associated with chondrodysplasia, a short‐legged phenotype that defined at least 19 dog breeds which include dachshund, corgi, and basset hound (Parker et al., 2009). These results indicate that a single gene can play critical roles in constraining and directing phenotypic diversity in the dog. However, there are very few reports about the sustainable evolution and innovative functions of new genes in the dog.

In this study, we used dog genome sequence, protein sequence, and ESTs data to identify many retrogenes and chimerical retrogenes that originated ~22 million years ago (Mya) after the divergence time between dog and its closely related species red fox. By analyzing their selective pressure and functions, we found that new retrogenes were likely available for improving dogs' adaptation to scavenge human waste dumps. Furthermore, we also detected a biased movement of functional retrogenes on and off the X chromosomes, which was likely driven by natural selection or sexual antagonism. This study will not only increase our understanding that new retrogenes can reshape the dog genome but also allow further exploration of the molecular mechanisms underlying the dogs' adaptive evolution.

## 2 MATERIALS AND METHODS

### 2.1 Identification of dog retrogenes

**Figure 2:**
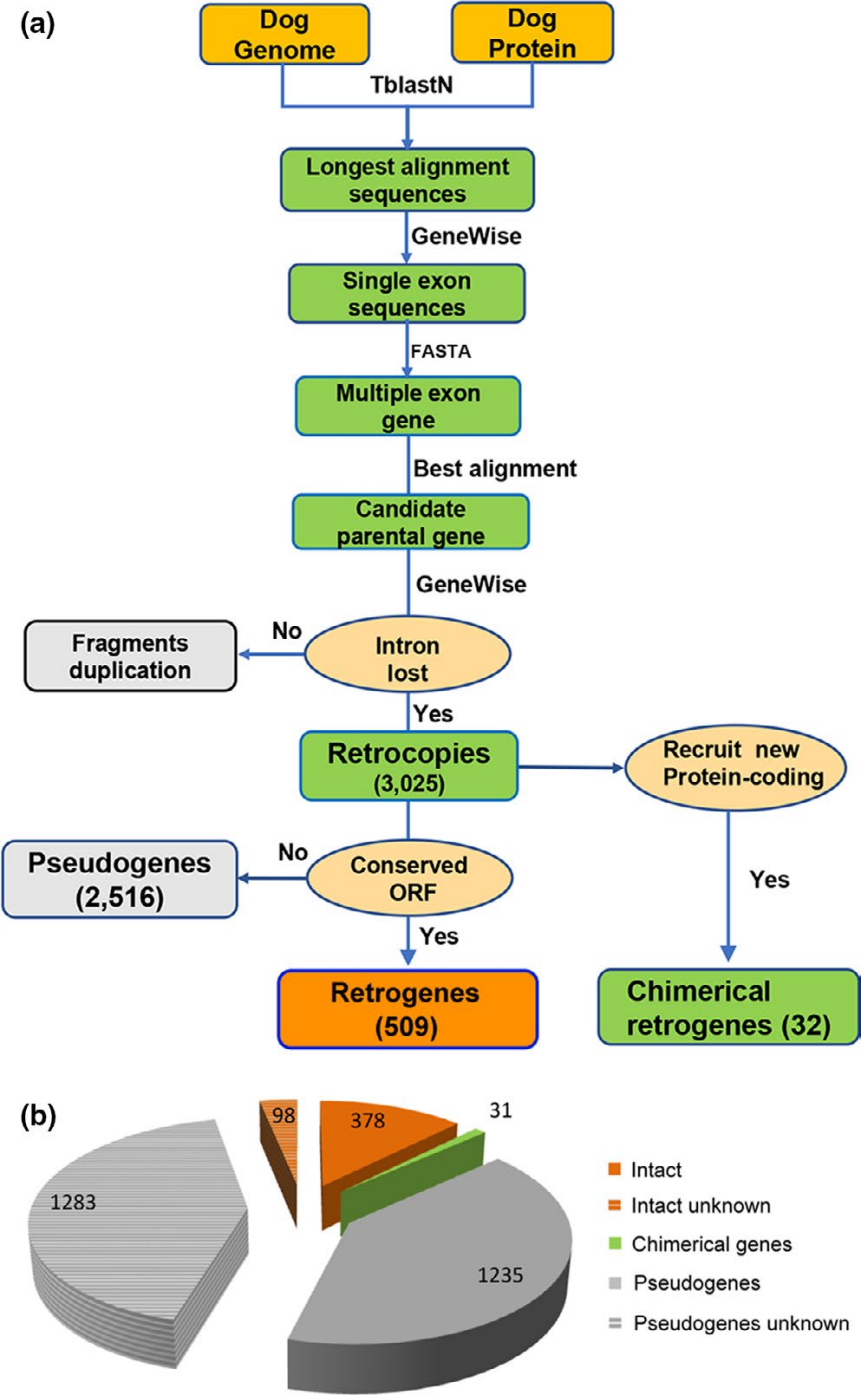
Identification of dog retrogenes. (a), The main procedure for identifying retrogenes. (b), Identification of different categories of retrocopies

To identify parental–retrogene pairs in the dog, we used a similar algorithm that was presented in previous studies (Figure 2a; Fu, Chen, Zou, Long, & He, 2010; Marques et al., 2005). Dog genome sequence and all dog annotated peptide sequences were downloaded from the Ensembl database (Release 90). To detect retrogenes, we aligned the 25,882 annotated protein sequences to the whole dog genome sequence using TblastN with an *E*‐value threshold at 10^–3^. The homology sequences (with identity >50%, overlap >50% and a minimum length of 50 amino acids) were maintained for assurance of high‐quality alignments. GeneWise software was used to define the intron–exon boundary of the merged target sequences with default settings (score >35). The single exon sequences were considered as candidate copies. Then, the FASTA software was used to perform similarity searches of candidate copies against all protein genes. If the best alignment of a single exon candidate copy was a protein gene with multiple coding exons, it was regarded as a candidate retrocopy. We then checked whether the introns of the parental gene (the best hit) had been lost in the retrocopies. If introns were retained, this retrocopy may be false positive and would be discarded. The retrocopies with either premature stop codons or frameshift mutations were defined as retropseudogenes. If one retrocopy could recruit novel regulatory elements and new protein‐coding exons and evolve into a functional retrogene, it was defined as a chimerical retrogene.

### 2.2 Divergence time and functionality analysis

We used the PAML35 package to calculate *K*_a_ (nonsynonymous substitution rate of nonsynonymous sites), *K*_s_ (synonymous substitution rate of synonymous sites), and *K*_a_/*K*_s_ (i.e., *ω*) between the parental gene and its retrocopy (Yang, 2007). According to the formula *T* = *K*_s_/2*λ* (*λ*: synonymous mutation rate), we estimated the origin time of retrocopies based on the *K*_s_ value. In addition, the *ω* value between paternal gene and retrogene pair was used to estimate functional constraints with *ω* < 1 representing the purifying selection, *ω* = 1 for the neutral selection, and *ω* > 1 for the positive selection. We applied a more stringent criterion (*ω* < 0.5) to examine the functional constraints of gene pairs. If *ω* value was <0.5, the gene was probably subjected to purifying selection and functional constraints.

### 2.3 Transcription analysis of the retrogenes

A total of dog ESTs were downloaded from the NCBI database and processed for various contaminants, low‐quality and low‐complexity sequences using the SeqClean program (https://sourceforge.net/projects/seqclean/files/). Then, the clean data were mapped to the dog genome with BLAT software. The alignments, with identity >95% and overlap >90%, were retained for further analysis. As we know, the new parental gene and retrogene pair generally shared a high identity in protein‐coding regions. Thus, the ESTs that were mapped to a unique location on the genome and with alignment sequence >100 bp and nucleotide identity of >97% were retained to analyze the transcription abilities of retrogenes.

### 2.4 GO enrichment and statistical analyses

Considering that the new parental–retrogene pair usually shared a high degree of identity in protein‐coding regions, it was viable to predict the retrogene's function through its corresponding parental gene's function. The dog annotation was relatively limited, so all genes were firstly converted into human homologous genes by HGNC symbol ID in the Ensembl BioMart database. Then, GO terms and pathway enrichment analyses were performed using Metascape (http://metascape.org) with the default parameters. And the top 20 significantly enriched clusters were reported (Benjamini–Hochberg corrected *q* < .05). The network graphs were edited using Cytoscape (Shannon et al., 2003). Gene movements among chromosomes were visualized by Circos software package (Krzywinski et al., 2009). All statistical analyses were carried out using the R programming language (version 3.2.0).

## 3 RESULTS AND DISCUSSION

### 3.1 Identification of dog retrogenes

**Figure 3:**
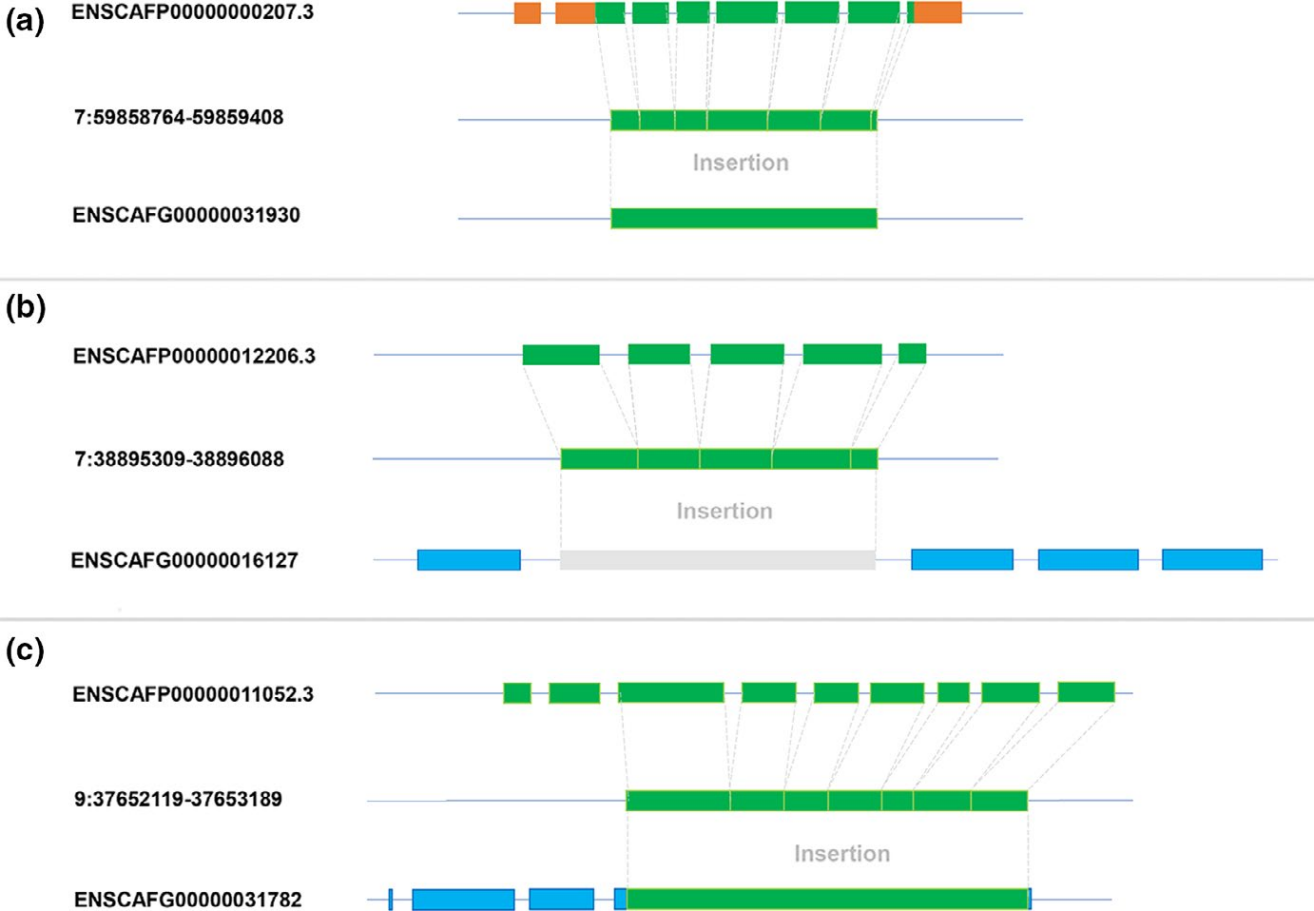
Three types of retrotransposition events. (a), (b), and (c) represent three types of retrotransposition events, respectively. Green boxes indicate coding sequence regions; orange boxes indicate UTR regions; blue boxes indicate new recruited coding sequence regions; gray box indicates a retrocopy inserted into the intron region of another gene

The origin of new RNA‐based duplication genes has been a significant driver of organismal evolution (Chen et al., 2013; Long et al., 2013). The main principle for identifying retrogenes was to search gene pairs where one copy was a multiple coding exons gene, whereas the other copy was a single coding exon gene (Figure 2a). In the gene pair, the single coding exon gene was defined as a retrogene while the multiple coding exons gene was defined as its parental gene. First, a total of 25,157 annotated dog protein sequences were mapped onto the dog genome with Tblastn software. We obtained the longest alignment sequences that mapped to the proteins. Then, we used the GeneWise program to identify single exon sequences. Only 10,544 single exon sequences were retained as candidate retrocopies (probably encode single exon protein). Second, we performed similarity searches of the candidate sequences against all proteins using FASTA (Akram et al., 2011) to find their corresponding parental genes (multiple exon genes). The best alignment protein of the candidate sequence was considered as a candidate parental protein (with identity >50%, overlap >50%, and more than 50 amino acids). Third, we used GeneWise program once again to check the gene pair where the parental gene should contain multiple coding exons and the retrogene should be a single coding exon gene. For example, a multiple coding exons gene ENSCAFP00000012206.3 was involved in a retrotransposition event and integrated into a new DNA locus. It recruited novel regulatory sequences in the 5′ and 3′ flanking regions and then generated a new single exon gene ENSCAFG00000031930 (Figure 3a). This single exon gene was defined as a putative retrogene. Finally, we totally identified 3,025 candidate retrocopies which originated from 1,227 parental genes (Table S1), indicating that one parental gene could produce about 2.41 retrocopies at an average level.

**Figure 4:**
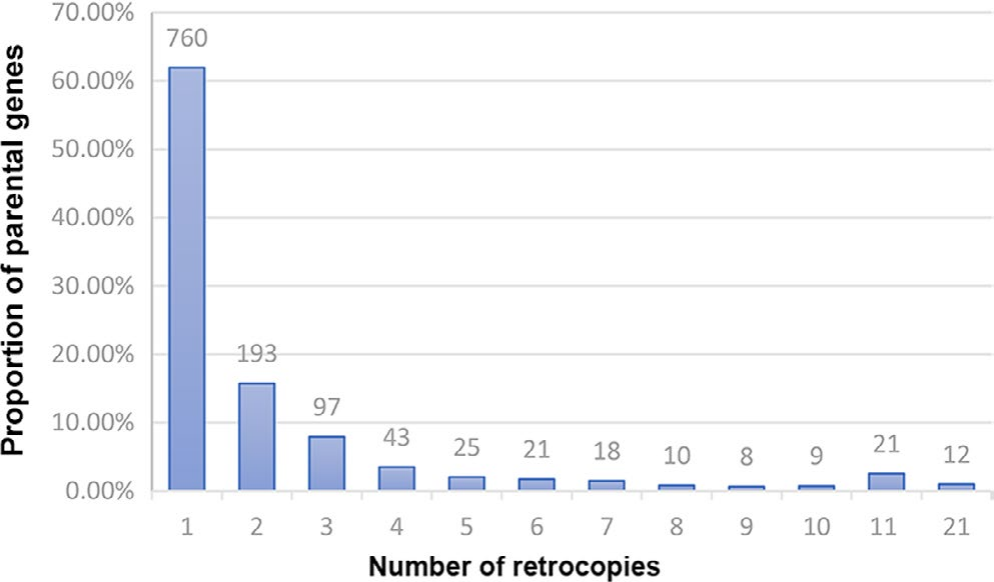
Distribution of retrocopy numbers

The distribution of retrocopy numbers (being produced by each parental gene) indicated that most of the parental genes (~62%, 760/1,227) could only generate one retrocopy, while a portion of parental genes could generate more than 10 retrocopies (Figure 4). Astonishingly, we analyzed the top 20 high‐yield parental genes and found that ENSCAFG00000005101 (RPSA) generated the most retrocopies ~41, followed by ENSCAFG00000017680 (RPL7A) producing 39 and ENSCAFG00000015077 (GAPDH) producing 37 (Table 1). Interestingly, the top 20 high‐yield parental genes were predominantly composed of ribosomal protein genes (60%, 12/20). For one thing, on average of 400 copies of rRNA genes existed in the mammalian genome and distributed in variable orientation on several chromosomes (Moss, Langlois, Gagnon‐Kugler, & Stefanovsky, 2007). High quantity of rRNA genes probably increased the opportunity of being involved in retrotransposition event. For another, the retrotransposon long interspersed element 1 (LINE1) composed the largest proportion of TE‐derived sequences. In embryos, LINE1 RNA could mediate binding of nucleolin and Kap1 to rDNA to regulate rRNA synthesis (Percharde et al., 2018). Thus, high‐response frequency with LINE1 would contribute to the formation of rDNA retrocopies.

**Table 1:** The top 20 high‐yield parental genes

Gene ID	Symbol	Retrocopy number
ENSCAFG00000005101	RPSA	41
ENSCAFG00000017680	RPL7A	39
ENSCAFG00000015077	GAPDH	37
ENSCAFG00000008021	RPL7	37
ENSCAFG00000001615	RPS6	33
ENSCAFG00000019472	RPS2	31
ENSCAFG00000005764	RPL15	31
ENSCAFG00000001818	DNAJA1	29
ENSCAFG00000006580	HNRNPA1	28
ENSCAFG00000008640	TUBA1B	25
ENSCAFG00000001318	RPL10A	23
ENSCAFG00000006597	HMGB1	21
ENSCAFG00000020136	RPL12	20
ENSCAFG00000008212	BTF3	20
ENSCAFG00000007990	RPS3A	19
ENSCAFG00000012195	LDHB	18
ENSCAFG00000032728	/	17
ENSCAFG00000003631	RPS11	16
ENSCAFG00000008873	RPL6	15
ENSCAFG00000018174	RPS15A	14

Note

/ represents no annotation gene.

Generally, the retrocopy only maintains the coding region of its parent gene and lose the corresponding regulatory elements such as promoters and enhancers, thus leading to accumulation of nonsense mutations and eventually evolving into a pseudogene. To obtain the expression ability, it should recruit new regulatory elements nearby to become a functional transcriptional gene. According to our GeneWise analysis, only ~16.8% (507/3,025) retrocopies retained their parent genes' open reading frame (ORF) sequence, which was considered as the “intact retrogenes.” The remaining 2,518 retrocopies were defined as pseudogenes as they exhibited either frameshift mutations or premature termination codons. This result was nearly equivalent to the proportion of intact retrogenes in human and rat genome (Kabza, Ciomborowska, & Makalowska, 2014; Marques et al., 2005). However, the total number of retrocopies in the dog was much higher than that in most other animals. One possibility was that a large number of LINEs and SINEs were observed to be overactive in the dog (Mamedov, Arzumanyan, Amosova, Lebedev, & Sverdlov, 2005; Wang & Kirkness, 2005). LINEs and SINEs are the major sources of insertional mutagenesis such as retrotransposition events (Esnault, Maestre, & Heidmann, 2000; Gogvadze & Buzdin, 2005; Schmid, 1998). They can increase the variability and instability of the dog genome and led to generate higher content of retrocopies. Even so, the majority of these retropseudogenes will be gradually eliminated during the evolutionary process in organisms, while a small part of them evolve into new functional genes by producing truncated protein or expressed lncRNA (Chen et al., 2013; Hirotsune et al., 2003; Podlaha & Zhang, 2004) and subsequently be retained under a long‐term natural selection.

If a retrocopy has inserted into the exon/intron structure of another gene (Figure 3b), it may disrupt the existing protein‐coding regions or genetic topology and result in deleterious mutations. Occasionally, a portion of retrocopies recruit new regulatory elements and protein‐coding exons nearby and then evolve into functional chimerical retrogenes. For example, ENSCAFG00000031782 was originated as insertion of the retrotransposition sequence of ENSCAFP00000011052.3 and recruited nearby novel regulatory sequences and protein‐coding regions to form a chimerical structure (Figure 3c). Out of 507 intact retrogenes, we also identified 31 chimerical retrogenes. By recruiting new protein‐coding regions, chimerical retrogenes are likely to evolve into novel protein genes and drive genetic innovation and adaptive evolution (Long et al., 2003). These 31 chimerical retrogenes were regarded as intact retrogenes in the following analysis.

In Ensembl release 90, the dog genome annotation GTF file contained ~32,704 genes and ~39,074 transcripts. In contrary, in our retrocopies data set, we detected more than 1,300 retrocopies that did not overlap with any annotated gene, suggesting that they were likely newly annotated genes or nonfunctional fragments (Figure 2b). Nevertheless, 98 of them were intact retrogenes and maintained the conserved ORF sequences of their parental genes. This result indicated that newly annotated retrogenes could not only shape the dog genome but also complement the blank position without annotation.

### 3.2 A burst of new retrogenes and chimerical retrogenes in the dog genome

**Figure 5:**
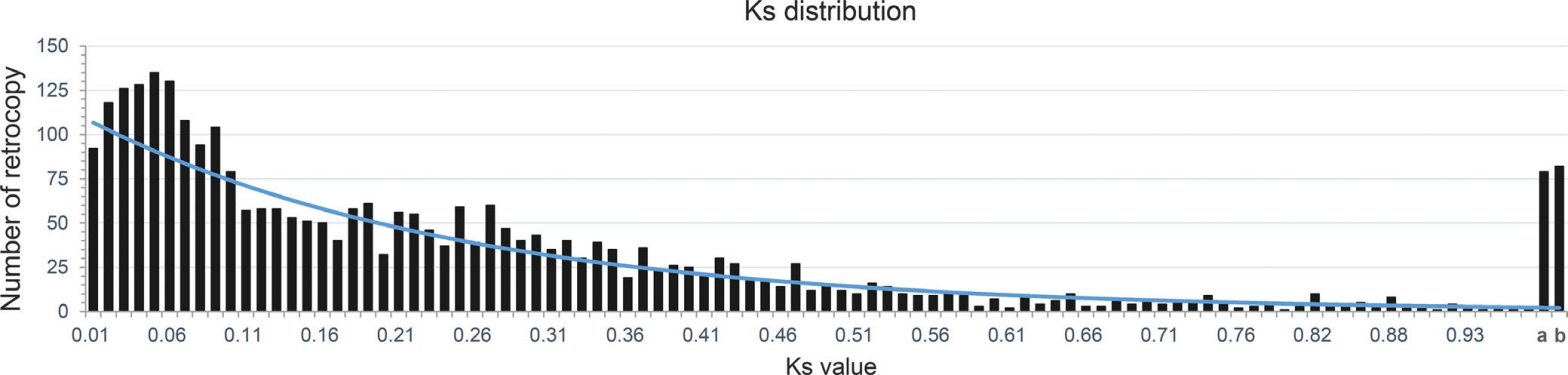
*K*_s_ distribution for 3,025 retrocopies. Retrocopies with *K*_s_ between 1.0–2.0 and ≥2.0 were, respectively, pooled in a single bin. a, with *K*_s_ between 1.0–2.0; b, with *K*_s_ ≥ 2.0. The peak of *K*_s_ in the range between 0.02–0.06 revealed a burst of retrotransposition in dog genome and mirrored the divergent time between dog and its closely related species red fox (estimated in http://www.timetree.org/, about 7.5–22.5 Mya)

In the dog, neutral mutation rate is estimated ~0.4 × 10^–8^ per generation (Frantz et al., 2016; Skoglund et al., 2015). We used a 3‐year generation time (corresponding to 1.33 × 10^–9^ mutations per year) to calculate the divergence time between retrocopies and corresponding parental genes (*T* = *K*_s_/2*λ*). The *K*_s_ distribution of retrocopies showed that a burst of retrotransposition (729, 24%) reached its peak region in the range of *K*_s_ 0.02–0.06 (Figure 5), approximately 7.5–22.5 million years ago (Mya), suggesting that a large number of new retrogenes originated during a short evolutionary history. Interestingly, the burst of retrocopies was corresponding to the divergence time between dog and its closely related species red fox (7.0–22.0 Mya from http://www.timetree.org/), implying that the retrotransposition was likely to drive the origin of new genes and consequently contributed to their differentiation or phenotypic traits. Consistent with previous studies in other mammals (Marques et al., 2005; Pan & Zhang, 2009), fish (Fu et al., 2010), and plant (Wang et al., 2006), this result confirmed that retrotransposition could play significant roles in both genome remolding and lineage or species differentiation.

Interestingly, we detected 14 intact retrogenes with *K*_s_ values of 0 which might be indicative of very recent retrogenes. And further investigation of these genes might shed light on new retrogenes which might be involved in dog‐specific phenotypic traits. Nevertheless, this study was entirely based on the analysis of the genome of domestic dog and the origin time of new retrogenes was estimated by *K*_s_ value. Thus, a further phylogenetic analysis, by comparing the other genomes such as the gray wolf and the red fox (Gopalakrishnan et al., 2017; Kukekova et al., 2018), would be helpful to validate dog‐specific or *Canini*‐specific candidate retrogenes. Function analysis of these dog‐specific new retrogenes would be highly valuable in revealing the molecular mechanism of artificial domestication.

In addition, we calculated the *K*_s_ distribution of 31 chimerical retrogenes and estimated their divergence time (Table S1). Interestingly, nine of them had *K*_s_ values lower than 0.06, implying that these nine new chimerical retrogenes formed in the last 22.5 million years (~0.4 chimerical retrogene per million years). The rate of formation of new chimerical retrogenes in dogs was more than three times higher than that in humans (~0.14 chimerical retrogenes per million years) while lower than the proportion of new chimerical retrogene in zebrafish (Fu et al., 2010) and rice (Wang et al., 2006). These results also indicated that the formation of dog chimerical retrogenes occurred at a remarkably rapid evolutionary rate.

### 3.3 The functionality of retrogenes

We applied a two‐step strategy to survey the functionality of retrocopies: (a) to compare nonsynonymous (*K*_a_) and synonymous (*K*_s_) substitution rates (*ω* ratios) between retrogenes and their parental genes; (b) to estimate the transcriptional ability of retrocopies.

**Figure 6:**
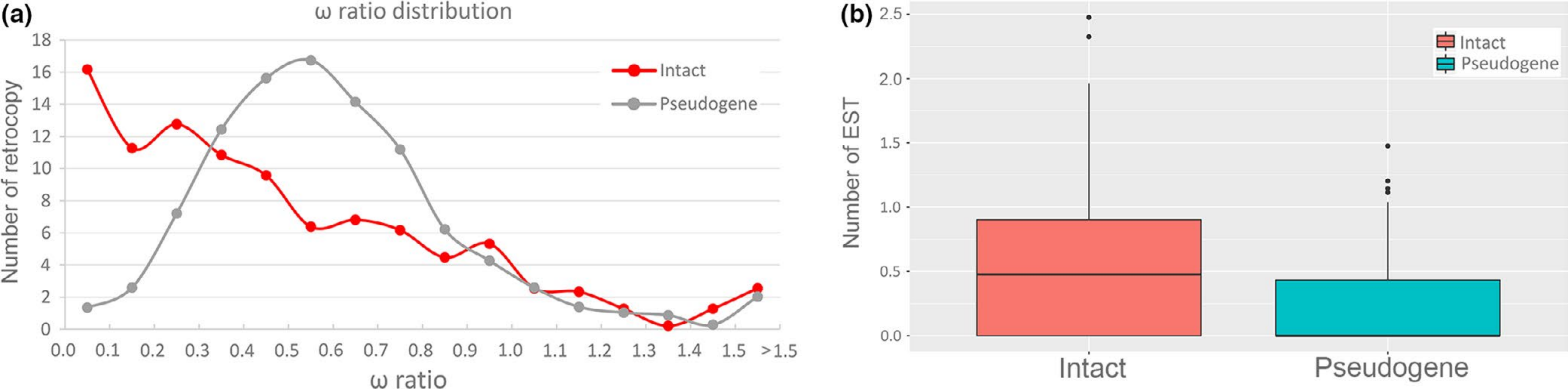
Functional analysis of retrogenes. (a), distribution of *ω* ratio between intact retrogenes and retropseudogenes. Retrocopies with *ω* > 1.5 were pooled in a single bin. (b), Transcription ability of intact retrogenes and retropseudogenes supported by ESTs

First, we tested the functional constraints at the protein level between parental genes and retrocopies by using the nonsynonymous to synonymous substitution rate (*ω* = *K*_a_/*K*_s_). The values *ω* < 1, *ω* = 1, and *ω* > 1 represent purifying selection, neutral evolution, and positive selection, respectively (Yang & Nielsen, 2002). We used a stricter criterion (*ω* < 0.5) to estimate the functionality of retrogenes (Emerson, Kaessmann, Betran, & Long, 2004). After removing 46 retrocopies with *K*_s_ = 0 (*ω* value is unable to evaluate), we obtained 470 intact retrogenes and 2,509 retropseudogenes for further analysis. Obviously, intact retrogenes significantly showed much lower *ω* ratio values than that in retropseudogenes (Figure 6a, *p* < .01). About 60.6% (285/470) of intact retrogenes had lower *ω* than 0.5, compared with 39.2% (984/2,509) of retropseudogenes (Table 2). Thus, these results indicated that more than half of intact retrogenes were under stronger purifying selection and tended to be subjected to more functional constraints when compared to retropseudogenes. This revealed a similar proportion of functional constraint in pig retrocopies (~64.7% of intact retrogenes and ~39.6% of retropseudogenes were subjected to functional constraint; Fang et al., 2018), suggesting that intact retrogenes were more likely to evolve into functional genes and subsequently be reserved by natural/artificial selection in mammals during a long evolutionary history. In addition, we detected about 25 retrogenes with *ω* ratios significantly higher than 1, suggesting that they were likely reserved by positive selection and evolved into novel functions. New genes have been considered to be an important driver to phenotypic evolution innovation (Kaessmann, 2010). They evolve important genetic novelties to facilitate biological diversity and contribute to the evolution of a lineage or species‐specific phenotypic traits (Chen et al., 2013, 2010; Long et al., 2013). Thus, dog retrogenes may play important roles in promoting adaptive evolution and species differentiation. Interestingly, a portion of retropseudogenes was under purifying selection or positive selection as well, suggesting that they were either function in splicing out the disabled mutations to produce novel proteins or had been functional but recently became pseudogenes.

**Table 2:** Selective pressure of retrocopies

Gene	*ω* < 0.5	*ω* = 0.5–1.2	*ω* > 1.2
Intact gene	285/470 (60.6%)	160/470 (34.1%)	25/470 (5.3%)
Retropseudogene	984/2,509 (39.2%)	1,419/2,509 (56.6%)	106/2,509 (4.2%)

Note

Intact gene: excluding 14 retrocopies with *K*_s_ = 0 and *ω* = NA; retropseudogene: excluding 1 retrocopy with *K*_s_ = 0 and *ω* = NA.

Moreover, transcription ability is another valuable way to validate gene functions. Considering that a retrogene shared high identity with its corresponding parental gene in coding regions, we used a more credible ESTs data to estimate the transcription ability instead of short‐tag expression sequences or hybridization‐based data (Fu et al., 2010; Harbers & Carninci, 2005). First, a total of 382,638 dog ESTs were downloaded from NCBI and processed for various contaminants such as vectors, adaptors, primers, or linker sequences using the SeqClean program. Then, about 375,093 clean ESTs were mapped onto the dog genome using BLAT software with default parameters. Only 242,251 high identity ESTs that aligned to a unique location in the dog genome were retained for further analyses. By analyzing the overlap region between retrocopy and ESTs, we found that intact retrogenes significantly exhibited much higher transcription ability than retropseudogenes (Figure 6b, *p* < .01). About 32.5% (165/507) of intact retrogenes were covered by at least one EST compared with only 5.0% (126/2,518) in retropseudogenes (Table 3). In the human genome, the proportion of expressed intact retrogenes has been reported about ~30.1% (Vinckenbosch, Dupanloup, & Kaessmann, 2006), indicating that a large proportion of retrocopies are transcribed and functionally reserved in mammals. In addition, 19 of 126 retropseudogenes were covered by more than four ESTs. Reading frameshifts were considered as one of the crucial molecular processes that could generate new gene structures. About 470 human gene duplicates had subjected to frameshift mutations and generate new coding sequences (Okamura, Feuk, Marques‐Bonet, Navarro, & Scherer, 2006). Thus, these 19 expressed retropseudogenes were likely functional transcript and performed reading frameshifts in a protein‐coding gene to generate novel proteins. This result supported the previous' result that a portion of retropseudogenes was subjected to stringent functional constraints.

**Table 3:** ESTs support of retrocopies

Raw ESTs	Clean ESTs	Uniq ESTs	Intact	Retropseudo
382,638	375,093	242, 251	165/507	126/2,518

Note

165/507 indicates that 165 intact retrogenes were supported by at least one.

### 3.4 New retrogenes improved dogs' adaptive evolution

**Figure 7:**
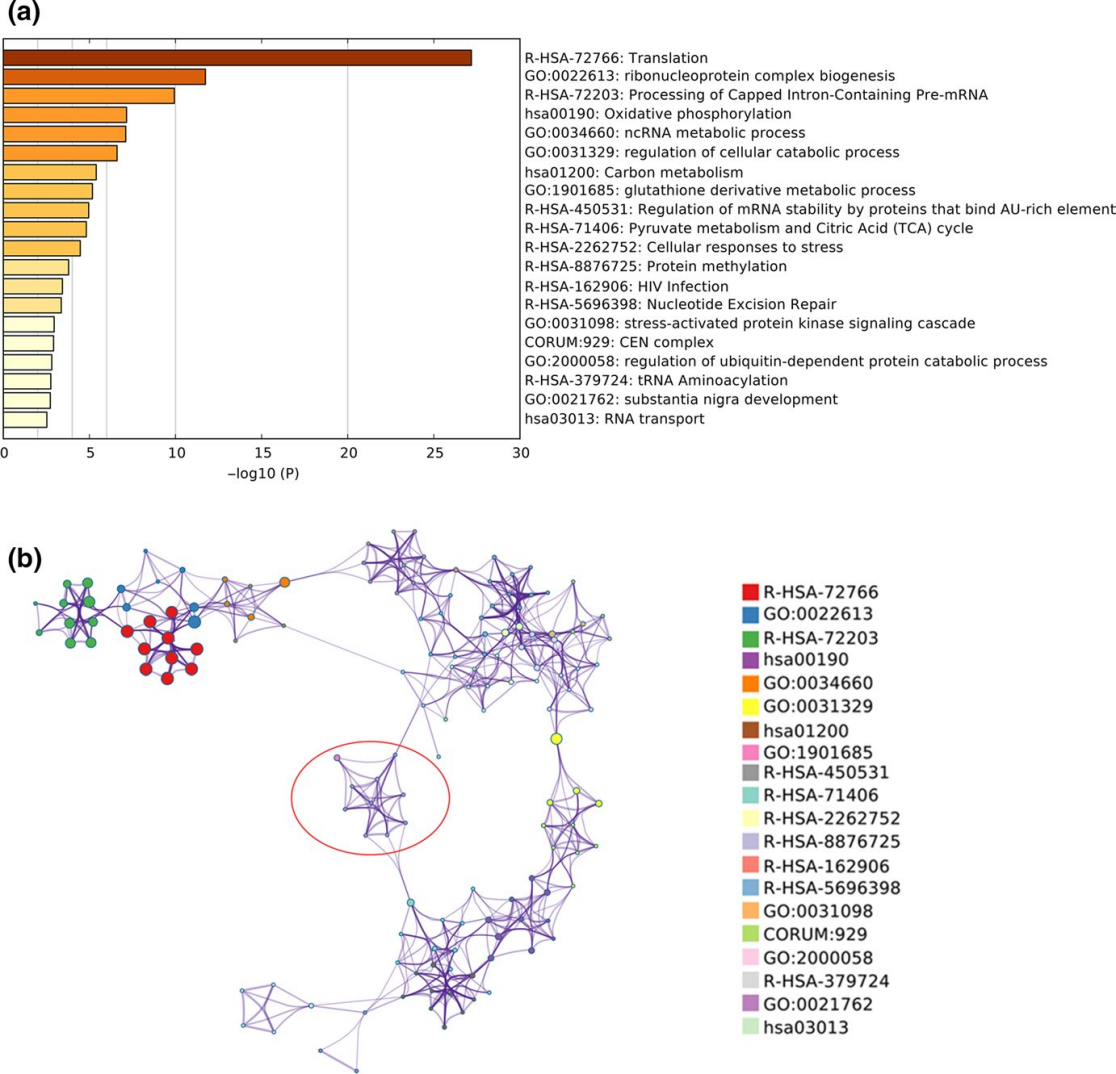
GO and pathway enrichment analysis of new retrogenes. (a), Heatmap of enriched terms of new retrogenes. Only top 20 enrichment with *p* < .01 are listed. (b) Network of enriched terms by cluster ID, where nodes that share the same cluster ID are typically close to each other. Glutathione biosynthetic/metabolic process and response to toxic substance is enriched in the red circle

Origin of new genes provides critical genetic novelties for biological diversity and contribute to the evolution of lineage‐ or species‐specific phenotypic traits (Betran, Wang, Jin, & Long, 2002; Chen et al., 2013, 2010). As a domestic species, the dog is a part of the largest groups of phenotypic diversity (Beck, 2002). We performed functional enrichment analyses of 207 new retrogenes (*K*_s_ < 0.06 and *ω* < 0.5) to investigate their contributions to dogs' adaptive evolution. Top 20 clusters with their representative enriched terms from Metascape pathway enrichment were performed with the following ontology sources: KEGG Pathway, GO Biological Processes, Reactome Gene Sets, and CORUMA (Figure 7a). The most significant GO terms mainly consisted of biological processes which were involved in ribonucleoprotein complex biogenesis, ncRNA metabolic process, regulation of cellular catabolic process, and glutathione derivative metabolic process. Moreover, pathways enrichment analysis was mainly involved in translation, oxidative phosphorylation, carbon metabolism, and RNA transport. Interestingly, we found that some new retrogenes were significantly enriched in glutathione biosynthetic/metabolic process (GSTA4, GSTP1, MGST3, AKR1A1, GPX8, and EEF1G) and response to toxic substances (ACTB, CCNB1, GOT2, GSTP1, HNRNPA1, MGST3, RPL10A, TXNL1, and GPX8; Figure 7b). For example, glutathione S‐transferases (GSTs, including GSTA4, GSTP1, and MGST3) was defined as the most important intracellular nonenzymatic antioxidant for their ability to catalyze the conjugation of the reduced form of glutathione (GSH) to xenobiotic substrates. GSTs exist extensively in plants and animals (Pompella, Visvikis, Paolicchi, De Tata, & Casini, 2003; Sies, 1999) and play critical roles in preventing damage to important cellular components that are caused by reactive oxygen species such as free radicals, peroxides, methylglyoxal, and lipid peroxides (Aoyama & Nakaki, 2015). It also effects in the detoxification of a variety of xenobiotics such as chemical carcinogens, environmental pollutants, and antitumor agents, which interact with glutathione and are ultimately excreted in the urine or feces in the form of mercapturic acids (Hernandez et al., 2015). During the early phase of the agricultural revolution, humans changed from a nomadic lifestyle to a sedentary lifestyle and ancestral wolves may have been attracted by dumps and spoiled food sources near early human settlements. Deterioration of food, especially lipids and proteins in the meat, could give rise to infectious organisms or toxic products such as bacteria, viruses, and lipid peroxidation. Thus, new genes that involved in glutathione biosynthetic/metabolic process and response to toxic substances pathways were likely helpful for wolves/dogs to detoxify environmental pollutants in intestinal. These genetic novelties allowed wolves/dogs groups for efficient use of dumped food by human and probably contributed to their adaptation to early human settlements. This is one possibility why ancestors of wolves/dogs were kept by the human.

In addition, we checked the transcription ability of these new retrogenes that significantly enriched in glutathione biosynthetic/metabolic process and response to the toxic substance. These 12 parental genes totally produced about 95 retrocopies (Table S2), of which 13 were defined as functional new retrogenes (with *K*_s_ < 0.06 and *ω* < 0.5). Moreover, eight functional new retrogenes were supported by at least one ESTs, suggesting that they were likely expressed and contributed to detoxify environmental pollutants in intestinal.

### 3.5 Extensive gene traffic on the X chromosome

Mammalian sex chromosomes (X and Y) change profoundly in their differentiation from ancestral autosomes. Throughout this process, the selective relocation of new genes can be driven by gene duplication (Emerson et al., 2004; Vibranovski, Zhang, & Long, 2009). Retrotransposition reshapes genome through the reverse transcription of mRNA and insertion of DNA into a new locus. Due to the distinguishing features between a retrocopy and its ancestral parental gene, it is feasible to identify the direction of the RNA‐duplicated gene in chromosomal movement. To elucidate gene movements and chromosome bias in the dog genomes, we analyzed the relocation distribution of retrogenes and their parental genes. By screening 3,025 retrotransposition events, 2,902, 77, and 46 of which were involved into interchromosomal, intrachromosomal, and scaffold/MT movements, respectively (corresponding to 95.93%, 2.55%, and 1.52%, respectively, Table S1), suggesting that retrocopies were inclined to distribute in new chromatin environments.

**Figure 8:**
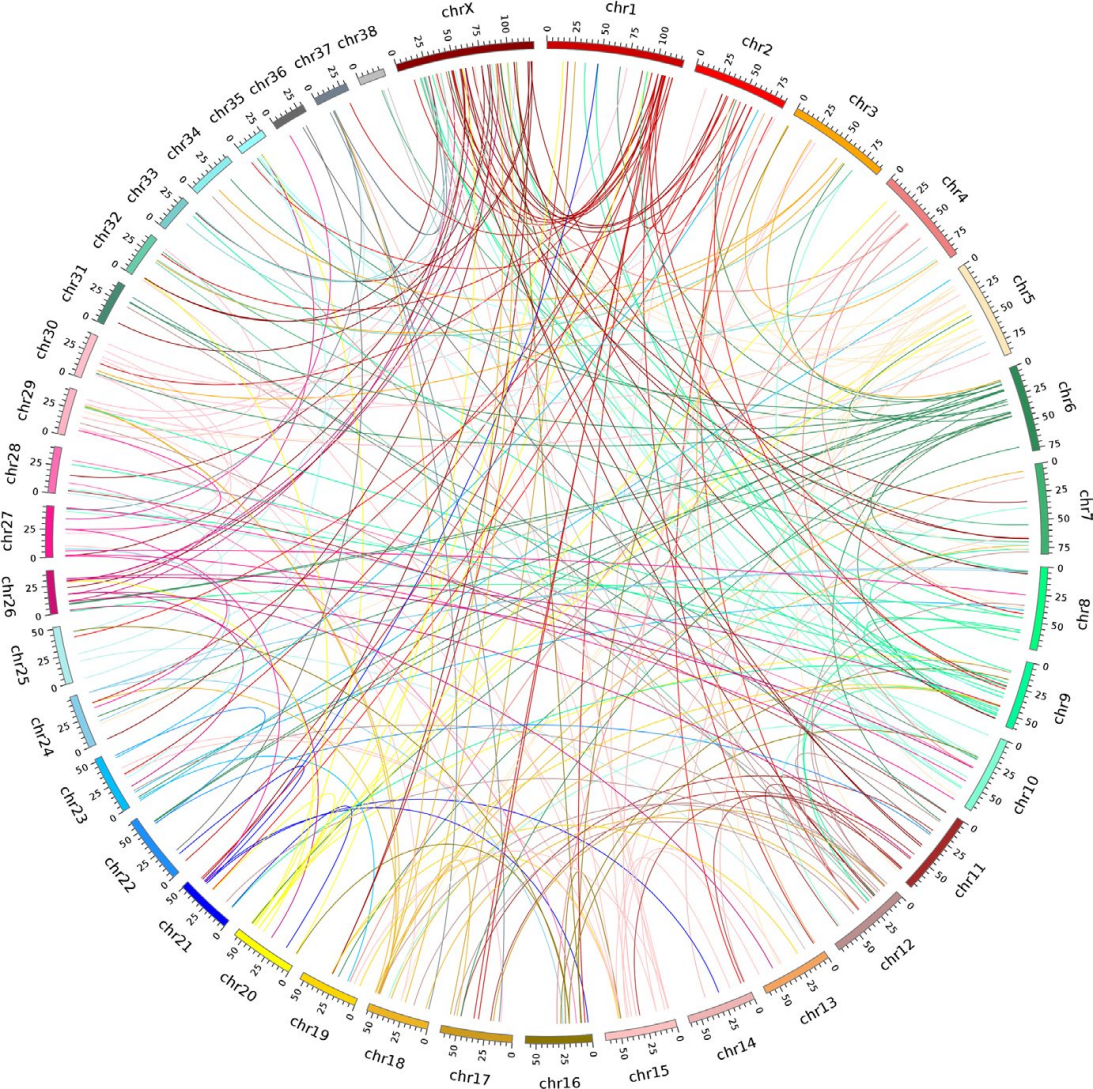
Gene interchromosomal movement. Different color bars represent different chromosomes. The line indicates gene interchromosomal movement, and its color indicate the movement direction

**Figure 9:**
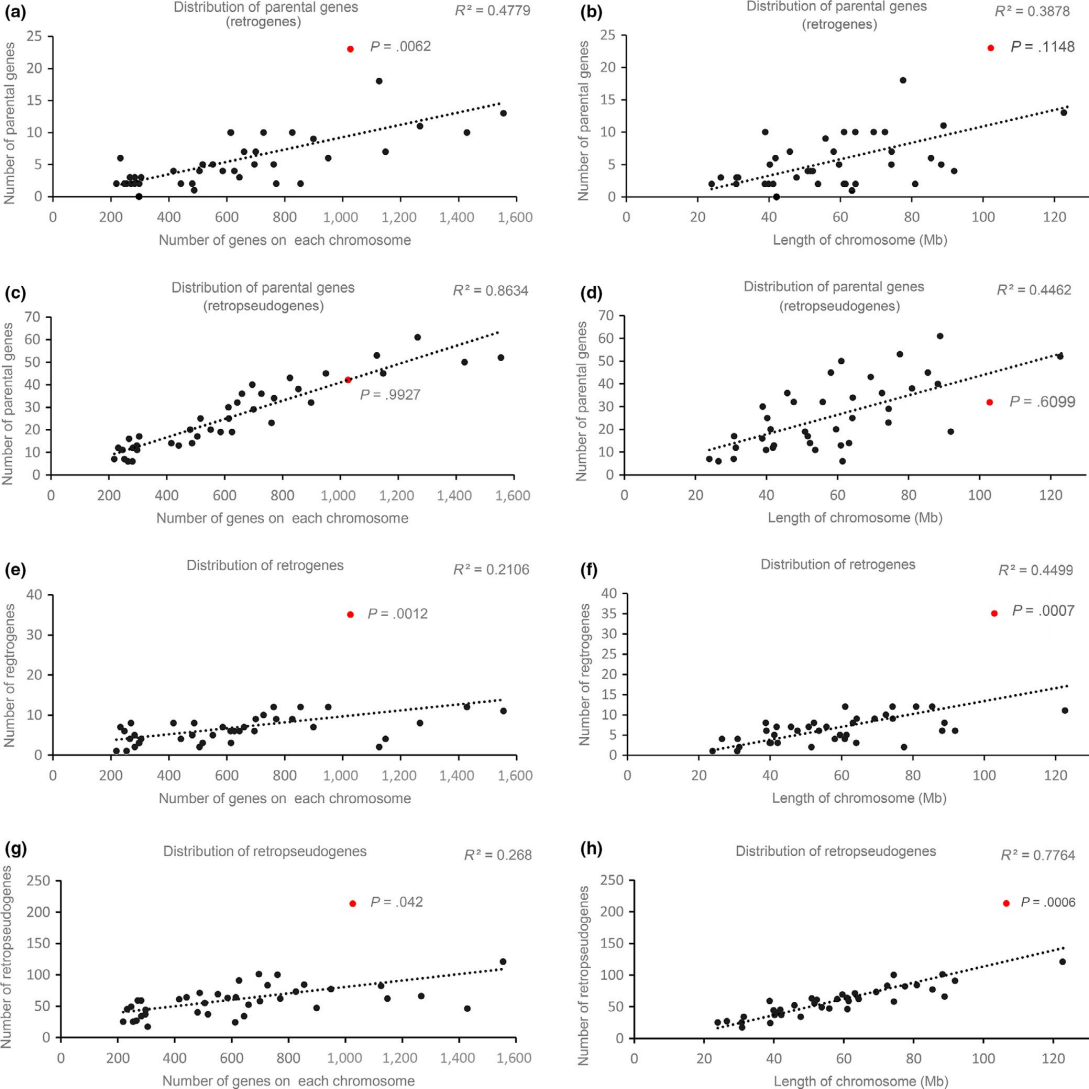
Analysis of retrocopies relocations. We estimate the relationship between genes movement bias and chromosomal characteristics (including the number of genes on each chromosome and the size of chromosomal). Regressions for the parental genes of retrogenes (a, b) and retropseudogenes (c, d). Regressions for retrogenes (e, f) and retropseudogenes (g, h). Red point represents X chromosome, and *p* < .01 indicates a significant excess movement our of/into X chromosome. *R* value represents the related coefficient

First, we estimated the relationship between functional retrogenes' movement bias and chromosomal characteristics (including the number of genes on each chromosome and the size of chromosomal). To avoid overestimating the number of functional retrogenes, we performed a stringent functionality criterion based on both selective constraints, with *ω* < 0.5, and conserved ORF region without frameshift mutations or premature stop codons. In total, we identified 272 functional retrogene pairs where the retrogenes did not share chromosome linkage with their parental copy (Figure 8). Analysis of the chromosomal location of their parental genes showed that the numbers of parental genes on each chromosome were positively associated with both the number of genes (*r* = .69, Figure 9a) and the size of chromosomes (*r* = .62, Figure 9b). One possibility was the case that the longer chromosomes usually contained more genes, which provided greater opportunities for being involved into retrotransposition event. However, as the only outlier, the X chromosome exhibited a significant excess of parental genes when compared with the autosomes (red dot, *p* = .0062, Figure 9a), indicating that parental genes escaped from X chromosome to autosomes. Previous study indicated that retrotransposition events occurred randomly between chromosomes (Zhang, Harrison, & Gerstein, 2002). Thus, we tested whether this asymmetric pattern of gene escaping event was consistent for nonfunctional retropseudogenes? To survey occasionality of abnormal movement out of the X chromosome into autosomes, we analyzed neutral patterns of parental gene distribution by using their nonfunctional retropseudogenes (0.5 < *ω* < 1.0, and with frameshift mutations or premature stop codons). A total of 1,268 retropseudogenes and parental genes pairs, which were involved in interchromosomal movements, were used to study the relationship between parental gene bias and chromosomal characteristics. Consistently, it was observed that the numbers of retropseudogenes' parental genes on each chromosome were positively associated with both the number of genes and the length of chromosome (*r* = .93, Figure 9c and *r* = .67, Figure 9d) and the X chromosome did not show significant different gene movements (red dot, *p* = .9927 and *p* = .6098). These results indicated that an excess of escaping event from the X chromosome to the autosomes was a widespread mechanism of how functional RNA‐duplicated genes originated and survived. In agreement with previous studies in human, mouse, and Drosophila (Betran, Thornton, et al., 2002; Emerson et al., 2004), the excess of parental genes escaping from the X chromosome to the autosomes may be explained by natural selection or sexual antagonism (Charlesworth, Coyne, & Barton, 1987; Engelstadter & Haig, 2008; Vibranovski et al., 2009; Wu & Xu, 2003). In the male germline, expression of parental genes located on inactivation X chromosome will be inhibited. By producing retrogenes in autosomes, the male‐biased genes avoid X‐linkage inactivation and become expressed genes in testis. Retrogenes that escaped from the X chromosome have a higher potential to be expressed in testis and probably evolve into new genes beneficial for males. In the sexual antagonism hypothesis, X‐inactivation has been supposed to attain equal gene expression between males and females (dosage compensation; Charlesworth, 1978; Engelstadter & Haig, 2008). And these genes which were beneficial for males but costly for females are probably to be fixed on the autosomes. In addition, retrogenes that originated from the X chromosome usually exhibit testis expression patterns in *Drosophila* (Bai, Casola, Feschotte, & Betran, 2007; Betran, Thornton, et al., 2002), confirming that the fixation of male‐biased beneficial mutations on the autosomes is a long natural selection bias.

Second, we surveyed the distribution of retrocopies on the chromosome to test functional/nonfunctional retrocopies' insertion bias. For functional retrogenes, the number of retrocopies was positively correlative with the length of each chromosome (*r* = .67, Figures 8f and 9e). Consistent with the relocation of parental genes, the X chromosome was an outlier with an excess of retrogenes recruitment from autosomes (red dot, *p* = .0007, Figure 9f). For nonfunctional retropseudogenes, a similar relocation bias has been observed in the X chromosome (red dot, *r* = .88, *p* = .0006, Figure 9h). These results implied that the bias in disproportionate insertions of both functional and nonfunctional retrocopies existed for the X chromosome. One possibility was that the X chromosome was enriched twofold for L1 repetitive elements (Bailey, Carrel, Chakravarti, & Eichler, 2000), which probably contributed to a bias in extra insertions or fewer deletions of retrocopies.

## 4 CONCLUSION

In this study, we identified a large number of new retrogenes and chimerical retrogenes originated within ~22 Mya. By analyzing selective pressure along with ESTs expression, most of the intact retrogenes were significantly under stronger purifying selection and subjected to the more functional constraint when compared to retropseudogenes. Interestingly, new retrogenes likely provided important genetic basis for dogs' adaptation to scavenge human waste dumps. Furthermore, we also found a biased movement of functional retrogenes from the X to A chromosomes and A to X chromosomes, which were likely driven by natural selection or sexual antagonistic. Together, our analysis demonstrates that retrotransposition is an important mechanism that can reshape the dog genome and probably contribute to its adaptive evolution.

## CONFLICT OF INTEREST

The authors declare no conflicts of interest.

## AUTHOR CONTRIBUTIONS

X.G. and Y.L. performed the data analysis and wrote the first draft of the manuscript; A.A.A., Y.W., and H.C. reviewed and edited the manuscript; X.G., Y.W., and H.C. developed the original idea and supervised the study.

## Data Availability

All relevant data are within the paper and its Supporting Information files (Tables S1 and S2).
